# Supplementary material for: Intra-Sample Heterogeneity of Potato Starch Reveals Fluctuation of Starch-Binding Proteins According to Granule Morphology
Source: Plants (Basel). 2019 Sep 4;8(9):324. doi: 10.3390/plants8090324 (PMC6784226; doi:10.3390/plants8090324)
Supplement: Supplementary file 1 [file plants-08-00324-s001.zip › Sup/Figure S1.docx]

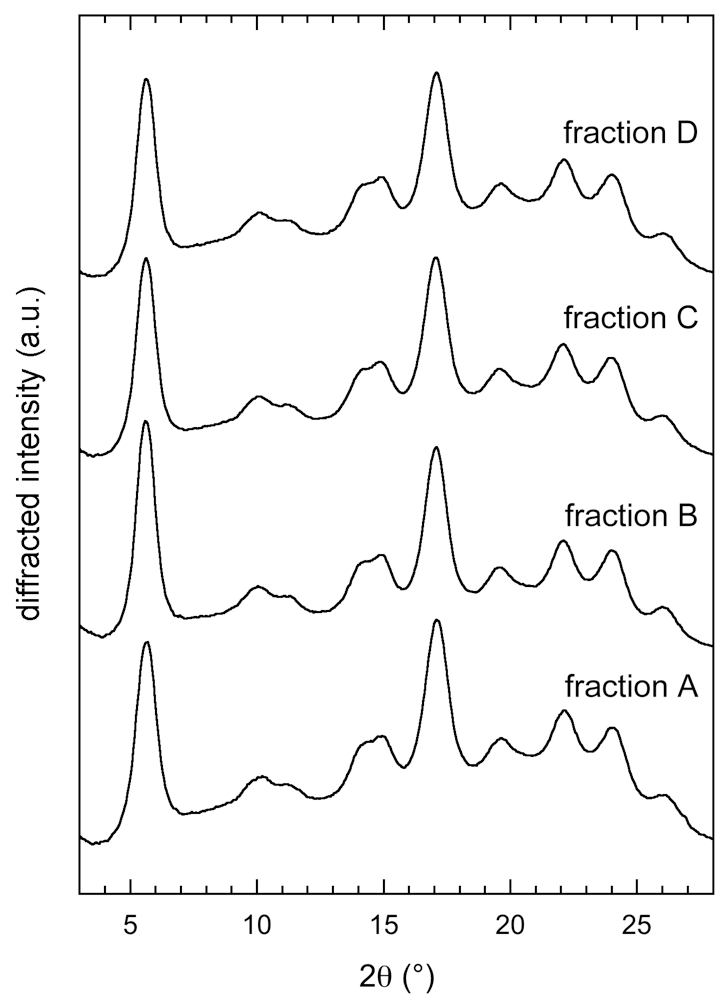


**Figure S1.** X-ray diffraction (XRD) profiles of size-fractionated Monalisa starch granules. Granules from fraction A had a diameter of 14.2 ± 3.7 µm, those from fraction B a diameter of 24.5 ± 6.5 µm; granules from fraction C and D had a diameter of 47.7 ± 12.8 and 61.8 ± 17.4 µm, respectively
