# Supplementary material for: Intra-Sample Heterogeneity of Potato Starch Reveals Fluctuation of Starch-Binding Proteins According to Granule Morphology
Source: Plants (Basel). 2019 Sep 4;8(9):324. doi: 10.3390/plants8090324 (PMC6784226; doi:10.3390/plants8090324)
Supplement: Supplementary file 1 [file plants-08-00324-s001.zip › Sup/Table S1.docx]

**Table S1.** Pearson paired-samples correlation analysis of starch-bound protein concentrations. The average concentrations in fmol.mg^-1^ were used for correlation analysis. Positive correlations with ρ > 0.95 and *p* < 0.05, ρ > 0.98 and *p* < 0.02, or ρ > 0.99 and *p* < 0.01 are highlighted in red. Significant negative correlation are highlighted in yellow with the use of the same thresholds.

|  | **GBSS** | **SS2** | **LESV** | **LSF2** | **THRx** | **GWD** | **SS3** | **CYP20.2** | **BE1.1** | **BE1.2** | **BE2** | **ESV1** | **PTST1** | **SS1** | **GPx** | **SEX4** | **SEX4.like** | **PHS1a** | **ISA3** | **PWD** | **SS4** | **PHS1b** | **SS6** |
| --- | --- | --- | --- | --- | --- | --- | --- | --- | --- | --- | --- | --- | --- | --- | --- | --- | --- | --- | --- | --- | --- | --- | --- |
| **GBSS** | 1,00 | 0,33 | -0,08 | 0,61 | 0,14 | -0,59 | 0,44 | 0,26 | 0,60 | 0,51 | 0,78 | -0,79 | 0,55 | 0,69 | 0,63 | 0,88 | 0,84 | 0,23 | 0,94 | -0,21 | 0,00 | -0,64 | 0,20 |
| **SS2** | 0,33 | 1,00 | 0,90 | 0,95 | 0,51 | -0,69 | 0,98 | -0,43 | -0,37 | -0,45 | 0,84 | 0,32 | 0,97 | 0,90 | -0,43 | 0,72 | 0,74 | 0,85 | 0,09 | 0,84 | -0,90 | 0,32 | -0,85 |
| **LESV** | -0,08 | 0,90 | 1,00 | 0,73 | 0,64 | -0,59 | 0,86 | -0,39 | -0,54 | -0,57 | 0,56 | 0,65 | 0,78 | 0,62 | -0,63 | 0,35 | 0,37 | 0,89 | -0,25 | 0,99 | -0,99 | 0,70 | -0,94 |
| **LSF2** | 0,61 | 0,95 | 0,73 | 1,00 | 0,50 | -0,79 | 0,97 | -0,25 | -0,10 | -0,19 | 0,97 | 0,00 | 1,00 | 0,99 | -0,13 | 0,90 | 0,90 | 0,80 | 0,40 | 0,64 | -0,76 | 0,06 | -0,64 |
| **THRx** | 0,14 | 0,51 | 0,64 | 0,50 | 1,00 | -0,87 | 0,63 | 0,45 | 0,23 | 0,23 | 0,50 | 0,15 | 0,53 | 0,37 | 0,09 | 0,23 | 0,16 | 0,89 | 0,24 | 0,58 | -0,73 | 0,64 | -0,35 |
| **GWD** | -0,59 | -0,69 | -0,59 | -0,79 | -0,87 | 1,00 | -0,81 | -0,36 | -0,36 | -0,30 | -0,84 | 0,17 | -0,78 | -0,72 | -0,26 | -0,67 | -0,60 | -0,89 | -0,59 | -0,48 | 0,68 | -0,25 | 0,31 |
| **SS3** | 0,44 | 0,98 | 0,86 | 0,97 | 0,63 | -0,81 | 1,00 | -0,25 | -0,20 | -0,27 | 0,90 | 0,19 | 0,99 | 0,92 | -0,26 | 0,77 | 0,77 | 0,91 | 0,25 | 0,79 | -0,89 | 0,30 | -0,76 |
| **CYP20.2** | 0,26 | -0,43 | -0,39 | -0,25 | 0,45 | -0,36 | -0,25 | 1,00 | 0,90 | 0,93 | -0,06 | -0,57 | -0,28 | -0,29 | 0,84 | -0,13 | -0,25 | 0,03 | 0,57 | -0,46 | 0,29 | -0,05 | 0,68 |
| **BE1.1** | 0,60 | -0,37 | -0,54 | -0,10 | 0,23 | -0,36 | -0,20 | 0,90 | 1,00 | 0,99 | 0,15 | -0,87 | -0,16 | -0,07 | 0,99 | 0,18 | 0,08 | -0,08 | 0,84 | -0,63 | 0,42 | -0,45 | 0,77 |
| **BE1.2** | 0,51 | -0,45 | -0,57 | -0,19 | 0,23 | -0,30 | -0,27 | 0,93 | 0,99 | 1,00 | 0,05 | -0,83 | -0,25 | -0,17 | 0,98 | 0,08 | -0,03 | -0,12 | 0,77 | -0,65 | 0,46 | -0,40 | 0,81 |
| **BE2** | 0,78 | 0,84 | 0,56 | 0,97 | 0,50 | -0,84 | 0,90 | -0,06 | 0,15 | 0,05 | 1,00 | -0,24 | 0,95 | 0,97 | 0,11 | 0,95 | 0,93 | 0,74 | 0,62 | 0,45 | -0,62 | -0,10 | -0,43 |
| **ESV1** | -0,79 | 0,32 | 0,65 | 0,00 | 0,15 | 0,17 | 0,19 | -0,57 | -0,87 | -0,83 | -0,24 | 1,00 | 0,08 | -0,11 | -0,92 | -0,41 | -0,35 | 0,29 | -0,89 | 0,75 | -0,57 | 0,82 | -0,76 |
| **PTST1** | 0,55 | 0,97 | 0,78 | 1,00 | 0,53 | -0,78 | 0,99 | -0,28 | -0,16 | -0,25 | 0,95 | 0,08 | 1,00 | 0,97 | -0,20 | 0,86 | 0,86 | 0,83 | 0,33 | 0,70 | -0,81 | 0,14 | -0,70 |
| **SS1** | 0,69 | 0,90 | 0,62 | 0,99 | 0,37 | -0,72 | 0,92 | -0,29 | -0,07 | -0,17 | 0,97 | -0,11 | 0,97 | 1,00 | -0,08 | 0,95 | 0,96 | 0,70 | 0,46 | 0,53 | -0,66 | -0,09 | -0,57 |
| **GPx** | 0,63 | -0,43 | -0,63 | -0,13 | 0,09 | -0,26 | -0,26 | 0,84 | 0,99 | 0,98 | 0,11 | -0,92 | -0,20 | -0,08 | 1,00 | 0,20 | 0,10 | -0,20 | 0,84 | -0,72 | 0,52 | -0,57 | 0,83 |
| **SEX4** | 0,88 | 0,72 | 0,35 | 0,90 | 0,23 | -0,67 | 0,77 | -0,13 | 0,18 | 0,08 | 0,95 | -0,41 | 0,86 | 0,95 | 0,20 | 1,00 | 0,99 | 0,51 | 0,69 | 0,23 | -0,40 | -0,38 | -0,29 |
| **SEX4.like** | 0,84 | 0,74 | 0,37 | 0,90 | 0,16 | -0,60 | 0,77 | -0,25 | 0,08 | -0,03 | 0,93 | -0,35 | 0,86 | 0,96 | 0,10 | 0,99 | 1,00 | 0,48 | 0,61 | 0,26 | -0,40 | -0,38 | -0,35 |
| **PHS1a** | 0,23 | 0,85 | 0,89 | 0,80 | 0,89 | -0,89 | 0,91 | 0,03 | -0,08 | -0,12 | 0,74 | 0,29 | 0,83 | 0,70 | -0,20 | 0,51 | 0,48 | 1,00 | 0,17 | 0,82 | -0,94 | 0,59 | -0,68 |
| **ISA3** | 0,94 | 0,09 | -0,25 | 0,40 | 0,24 | -0,59 | 0,25 | 0,57 | 0,84 | 0,77 | 0,62 | -0,89 | 0,33 | 0,46 | 0,84 | 0,69 | 0,61 | 0,17 | 1,00 | -0,38 | 0,14 | -0,59 | 0,44 |
| **PWD** | -0,21 | 0,84 | 0,99 | 0,64 | 0,58 | -0,48 | 0,79 | -0,46 | -0,63 | -0,65 | 0,45 | 0,75 | 0,70 | 0,53 | -0,72 | 0,23 | 0,26 | 0,82 | -0,38 | 1,00 | -0,97 | 0,76 | -0,96 |
| **SS4** | 0,00 | -0,90 | -0,99 | -0,76 | -0,73 | 0,68 | -0,89 | 0,29 | 0,42 | 0,46 | -0,62 | -0,57 | -0,81 | -0,66 | 0,52 | -0,40 | -0,40 | -0,94 | 0,14 | -0,97 | 1,00 | -0,69 | 0,89 |
| **PHS1b** | -0,64 | 0,32 | 0,70 | 0,06 | 0,64 | -0,25 | 0,30 | -0,05 | -0,45 | -0,40 | -0,10 | 0,82 | 0,14 | -0,09 | -0,57 | -0,38 | -0,38 | 0,59 | -0,59 | 0,76 | -0,69 | 1,00 | -0,60 |
| **SS6** | 0,20 | -0,85 | -0,94 | -0,64 | -0,35 | 0,31 | -0,76 | 0,68 | 0,77 | 0,81 | -0,43 | -0,76 | -0,70 | -0,57 | 0,83 | -0,29 | -0,35 | -0,68 | 0,44 | -0,96 | 0,89 | -0,60 | 1,00 |
