# Supplementary material for: Intra-Sample Heterogeneity of Potato Starch Reveals Fluctuation of Starch-Binding Proteins According to Granule Morphology
Source: Plants (Basel). 2019 Sep 4;8(9):324. doi: 10.3390/plants8090324 (PMC6784226; doi:10.3390/plants8090324)
Supplement: Supplementary file 1 [file plants-08-00324-s001.zip › Sup/Table S2.docx]

**Table S2.** Pearson paired-samples correlation analysis between starch-bound protein concentrations and the relative proportion of each DP from chain length distribution (CLD) analysis. The average protein concentrations in fmol.mg^-1^ and the relative proportions of DPs in % were used for correlation analysis. Positive correlations with ρ > 0.80 and *p* < 0.2, ρ > 0.98 and *p* < 0.02, or ρ > 0.99 and *p* < 0.01 are highlighted in red. Significant negative correlation are highlighted in yellow with the use of the same thresholds.

|  | **GBSS** | **SS2** | **LESV** | **LSF2** | **THRX** | **GWD** | **SS3** | **CYP20.2** | **BE1.1** | **BE1.2** | **BE2** | **ESV1** | **PTST1** | **SS1** | **GPX** | **SEX4** | **SEX4.like** | **PHS1a** | **ISA3** | **PWD** | **SS4** | **PHS1b** | **SS6** |
| --- | --- | --- | --- | --- | --- | --- | --- | --- | --- | --- | --- | --- | --- | --- | --- | --- | --- | --- | --- | --- | --- | --- | --- |
| **DP4** | 0,58 | 0,81 | 0,50 | 0,86 | 0,00 | -0,39 | 0,76 | -0,59 | -0,30 | -0,40 | 0,81 | -0,03 | 0,85 | 0,92 | -0,27 | 0,87 | 0,92 | 0,42 | 0,27 | 0,44 | -0,49 | -0,25 | -0,59 |
| **DP5** | 0,47 | 0,79 | 0,51 | 0,81 | -0,09 | -0,28 | 0,72 | -0,70 | -0,43 | -0,52 | 0,73 | 0,07 | 0,80 | 0,86 | -0,38 | 0,80 | 0,86 | 0,37 | 0,14 | 0,47 | -0,48 | -0,22 | -0,64 |
| **DP6** | 0,12 | 0,59 | 0,40 | 0,52 | -0,38 | 0,12 | 0,45 | -0,93 | -0,70 | -0,77 | 0,38 | 0,30 | 0,52 | 0,58 | -0,63 | 0,48 | 0,58 | 0,09 | -0,22 | 0,42 | -0,33 | -0,17 | -0,65 |
| **DP7** | 0,50 | 0,97 | 0,76 | 0,97 | 0,35 | -0,64 | 0,95 | -0,46 | -0,30 | -0,39 | 0,90 | 0,13 | 0,98 | 0,97 | -0,32 | 0,85 | 0,88 | 0,73 | 0,24 | 0,69 | -0,77 | 0,07 | -0,75 |
| **DP8** | 0,56 | 0,95 | 0,71 | 0,98 | 0,34 | -0,65 | 0,94 | -0,44 | -0,25 | -0,34 | 0,92 | 0,06 | 0,98 | 0,98 | -0,26 | 0,88 | 0,91 | 0,71 | 0,30 | 0,64 | -0,73 | 0,01 | -0,70 |
| **DP9** | 0,54 | 0,97 | 0,75 | 0,99 | 0,39 | -0,68 | 0,96 | -0,41 | -0,25 | -0,34 | 0,92 | 0,09 | 0,99 | 0,98 | -0,27 | 0,87 | 0,89 | 0,75 | 0,28 | 0,67 | -0,76 | 0,06 | -0,72 |
| **DP10** | 0,25 | 0,97 | 0,85 | 0,89 | 0,30 | -0,49 | 0,91 | -0,64 | -0,54 | -0,62 | 0,75 | 0,39 | 0,91 | 0,87 | -0,56 | 0,68 | 0,73 | 0,70 | -0,04 | 0,82 | -0,83 | 0,24 | -0,89 |
| **DP11** | -0,84 | -0,37 | -0,13 | -0,60 | -0,62 | 0,87 | -0,53 | -0,59 | -0,73 | -0,66 | -0,77 | 0,63 | -0,56 | -0,60 | -0,67 | -0,71 | -0,62 | -0,55 | -0,90 | 0,00 | 0,25 | 0,20 | -0,14 |
| **DP12** | -0,99 | -0,45 | -0,06 | -0,71 | -0,24 | 0,68 | -0,56 | -0,22 | -0,54 | -0,44 | -0,86 | 0,70 | -0,66 | -0,78 | -0,55 | -0,93 | -0,88 | -0,37 | -0,91 | 0,07 | 0,14 | 0,53 | -0,07 |
| **DP13** | -0,85 | -0,75 | -0,46 | -0,92 | -0,52 | 0,87 | -0,84 | -0,08 | -0,29 | -0,20 | -0,99 | 0,37 | -0,89 | -0,93 | -0,26 | -0,95 | -0,92 | -0,70 | -0,72 | -0,33 | 0,53 | 0,17 | 0,30 |
| **DP14** | -0,77 | -0,76 | -0,53 | -0,91 | -0,66 | 0,94 | -0,87 | -0,17 | -0,31 | -0,23 | -0,97 | 0,30 | -0,89 | -0,89 | -0,25 | -0,88 | -0,84 | -0,80 | -0,69 | -0,41 | 0,61 | 0,02 | 0,33 |
| **DP15** | -0,79 | -0,80 | -0,54 | -0,94 | -0,58 | 0,90 | -0,89 | -0,06 | -0,24 | -0,15 | -0,99 | 0,28 | -0,92 | -0,94 | -0,20 | -0,93 | -0,89 | -0,77 | -0,67 | -0,42 | 0,61 | 0,07 | 0,38 |
| **DP16** | -0,78 | -0,79 | -0,55 | -0,93 | -0,62 | 0,92 | -0,88 | -0,11 | -0,27 | -0,18 | -0,98 | 0,28 | -0,91 | -0,92 | -0,22 | -0,91 | -0,87 | -0,79 | -0,67 | -0,42 | 0,62 | 0,03 | 0,36 |
| **DP17** | -0,79 | -0,83 | -0,54 | -0,96 | -0,49 | 0,84 | -0,89 | 0,05 | -0,16 | -0,07 | -1,00 | 0,26 | -0,94 | -0,97 | -0,13 | -0,96 | -0,94 | -0,73 | -0,63 | -0,43 | 0,60 | 0,12 | 0,42 |
| **DP18** | -0,79 | -0,81 | -0,54 | -0,95 | -0,56 | 0,88 | -0,89 | -0,03 | -0,22 | -0,12 | -1,00 | 0,28 | -0,93 | -0,95 | -0,18 | -0,94 | -0,91 | -0,76 | -0,66 | -0,42 | 0,61 | 0,08 | 0,39 |
| **DP19** | -0,88 | -0,73 | -0,36 | -0,90 | -0,27 | 0,70 | -0,78 | 0,11 | -0,19 | -0,09 | -0,96 | 0,41 | -0,87 | -0,95 | -0,20 | -1,00 | -0,99 | -0,53 | -0,70 | -0,25 | 0,42 | 0,35 | 0,29 |
| **DP20** | -0,80 | -0,83 | -0,53 | -0,96 | -0,46 | 0,82 | -0,89 | 0,07 | -0,15 | -0,05 | -1,00 | 0,27 | -0,94 | -0,97 | -0,13 | -0,97 | -0,95 | -0,71 | -0,63 | -0,42 | 0,59 | 0,14 | 0,42 |
| **DP21** | -0,80 | -0,83 | -0,53 | -0,96 | -0,45 | 0,81 | -0,88 | 0,08 | -0,14 | -0,05 | -1,00 | 0,27 | -0,94 | -0,98 | -0,12 | -0,97 | -0,95 | -0,70 | -0,63 | -0,41 | 0,58 | 0,15 | 0,42 |
| **DP22** | -0,63 | -0,93 | -0,67 | -0,99 | -0,37 | 0,70 | -0,94 | 0,35 | 0,15 | 0,25 | -0,95 | 0,02 | -0,98 | -1,00 | 0,16 | -0,92 | -0,93 | -0,71 | -0,39 | -0,59 | 0,70 | 0,04 | 0,64 |
| **DP23** | -0,79 | -0,83 | -0,52 | -0,96 | -0,37 | 0,75 | -0,88 | 0,17 | -0,09 | 0,02 | -0,99 | 0,26 | -0,94 | -0,99 | -0,07 | -0,98 | -0,98 | -0,66 | -0,60 | -0,41 | 0,57 | 0,19 | 0,44 |
| **DP24** | -0,63 | -0,83 | -0,52 | -0,90 | -0,07 | 0,47 | -0,80 | 0,52 | 0,23 | 0,34 | -0,86 | 0,08 | -0,88 | -0,95 | 0,20 | -0,91 | -0,95 | -0,48 | -0,34 | -0,44 | 0,52 | 0,24 | 0,57 |
| **DP25** | -0,79 | -0,83 | -0,51 | -0,96 | -0,32 | 0,72 | -0,87 | 0,21 | -0,05 | 0,06 | -0,98 | 0,24 | -0,93 | -0,99 | -0,04 | -0,98 | -0,98 | -0,63 | -0,57 | -0,40 | 0,55 | 0,22 | 0,45 |
| **DP26** | -0,99 | -0,24 | 0,16 | -0,54 | -0,15 | 0,58 | -0,36 | -0,36 | -0,69 | -0,61 | -0,72 | 0,84 | -0,47 | -0,61 | -0,71 | -0,82 | -0,77 | -0,19 | -0,97 | 0,29 | -0,07 | 0,65 | -0,29 |
| **DP27** | -0,55 | -0,83 | -0,72 | -0,90 | -0,82 | 0,97 | -0,92 | -0,14 | -0,16 | -0,09 | -0,90 | 0,04 | -0,90 | -0,83 | -0,07 | -0,74 | -0,69 | -0,94 | -0,48 | -0,62 | 0,80 | -0,28 | 0,50 |
| **DP28** | -0,14 | -0,95 | -0,98 | -0,85 | -0,70 | 0,73 | -0,95 | 0,30 | 0,38 | 0,43 | -0,72 | -0,47 | -0,89 | -0,76 | 0,47 | -0,52 | -0,53 | -0,94 | 0,03 | -0,94 | 0,99 | -0,58 | 0,88 |
| **DP29** | 0,10 | 0,77 | 0,88 | 0,69 | 0,93 | -0,85 | 0,83 | 0,08 | -0,10 | -0,12 | 0,62 | 0,38 | 0,73 | 0,57 | -0,23 | 0,36 | 0,32 | 0,99 | 0,07 | 0,83 | -0,93 | 0,70 | -0,67 |
| **DP30** | 0,87 | 0,60 | 0,18 | 0,79 | -0,01 | -0,47 | 0,62 | -0,24 | 0,14 | 0,03 | 0,85 | -0,47 | 0,74 | 0,88 | 0,19 | 0,97 | 0,98 | 0,29 | 0,66 | 0,08 | -0,22 | -0,56 | -0,20 |
| **DP31** | 0,62 | 0,80 | 0,65 | 0,89 | 0,80 | -0,98 | 0,90 | 0,19 | 0,24 | 0,17 | 0,92 | -0,13 | 0,88 | 0,83 | 0,16 | 0,77 | 0,72 | 0,90 | 0,56 | 0,54 | -0,74 | 0,20 | -0,43 |
| **DP32** | 0,81 | 0,82 | 0,51 | 0,96 | 0,41 | -0,79 | 0,87 | -0,11 | 0,14 | 0,03 | 0,99 | -0,28 | 0,93 | 0,98 | 0,12 | 0,98 | 0,97 | 0,67 | 0,63 | 0,39 | -0,56 | -0,19 | -0,41 |
| **DP33** | 0,50 | 0,95 | 0,82 | 0,97 | 0,69 | -0,88 | 0,99 | -0,14 | -0,08 | -0,16 | 0,93 | 0,11 | 0,98 | 0,92 | -0,15 | 0,78 | 0,77 | 0,93 | 0,34 | 0,74 | -0,87 | 0,28 | -0,68 |
| **DP34** | 0,75 | 0,87 | 0,60 | 0,98 | 0,52 | -0,85 | 0,92 | -0,08 | 0,11 | 0,01 | 1,00 | -0,19 | 0,96 | 0,98 | 0,07 | 0,94 | 0,92 | 0,77 | 0,58 | 0,49 | -0,66 | -0,05 | -0,48 |
| **DP35** | 0,81 | 0,80 | 0,52 | 0,94 | 0,54 | -0,88 | 0,88 | 0,04 | 0,24 | 0,14 | 1,00 | -0,30 | 0,92 | 0,94 | 0,20 | 0,94 | 0,91 | 0,74 | 0,67 | 0,40 | -0,59 | -0,11 | -0,37 |
| **DP36** | 0,76 | 0,82 | 0,58 | 0,95 | 0,62 | -0,91 | 0,91 | 0,06 | 0,21 | 0,13 | 0,99 | -0,24 | 0,93 | 0,93 | 0,16 | 0,91 | 0,87 | 0,80 | 0,63 | 0,46 | -0,65 | -0,01 | -0,41 |
| **DP37** | 0,80 | 0,76 | 0,51 | 0,91 | 0,63 | -0,93 | 0,86 | 0,16 | 0,32 | 0,23 | 0,97 | -0,32 | 0,89 | 0,90 | 0,27 | 0,90 | 0,85 | 0,77 | 0,71 | 0,38 | -0,59 | -0,06 | -0,31 |
| **DP38** | 0,68 | 0,79 | 0,61 | 0,90 | 0,75 | -0,97 | 0,89 | 0,18 | 0,27 | 0,19 | 0,94 | -0,19 | 0,89 | 0,86 | 0,19 | 0,82 | 0,77 | 0,87 | 0,61 | 0,50 | -0,70 | 0,12 | -0,39 |
| **DP39** | 0,75 | 0,73 | 0,52 | 0,88 | 0,71 | -0,96 | 0,85 | 0,24 | 0,36 | 0,28 | 0,95 | -0,30 | 0,86 | 0,85 | 0,29 | 0,84 | 0,79 | 0,81 | 0,70 | 0,40 | -0,61 | 0,02 | -0,30 |
| **DP40** | 0,70 | 0,67 | 0,51 | 0,81 | 0,79 | -0,99 | 0,80 | 0,36 | 0,42 | 0,36 | 0,89 | -0,29 | 0,79 | 0,76 | 0,34 | 0,76 | 0,69 | 0,83 | 0,69 | 0,38 | -0,60 | 0,10 | -0,24 |
| **DP41** | 0,73 | 0,61 | 0,43 | 0,77 | 0,78 | -0,98 | 0,75 | 0,42 | 0,50 | 0,43 | 0,86 | -0,36 | 0,75 | 0,73 | 0,42 | 0,74 | 0,67 | 0,78 | 0,74 | 0,31 | -0,54 | 0,05 | -0,16 |
| **DP42** | 0,73 | 0,43 | 0,27 | 0,63 | 0,76 | -0,94 | 0,60 | 0,59 | 0,65 | 0,60 | 0,76 | -0,48 | 0,60 | 0,59 | 0,58 | 0,65 | 0,56 | 0,68 | 0,81 | 0,14 | -0,39 | -0,01 | 0,03 |
| **DP43** | 0,74 | 0,41 | 0,24 | 0,61 | 0,74 | -0,92 | 0,57 | 0,61 | 0,68 | 0,63 | 0,75 | -0,51 | 0,58 | 0,57 | 0,61 | 0,64 | 0,55 | 0,65 | 0,82 | 0,11 | -0,35 | -0,04 | 0,06 |
| **DP44** | 0,71 | 0,29 | 0,14 | 0,50 | 0,73 | -0,88 | 0,47 | 0,71 | 0,76 | 0,72 | 0,66 | -0,56 | 0,47 | 0,46 | 0,68 | 0,56 | 0,46 | 0,58 | 0,83 | 0,02 | -0,26 | -0,05 | 0,17 |
| **DP45** | 0,65 | 0,17 | 0,05 | 0,38 | 0,72 | -0,83 | 0,36 | 0,80 | 0,81 | 0,79 | 0,55 | -0,57 | 0,35 | 0,34 | 0,74 | 0,45 | 0,34 | 0,51 | 0,81 | -0,06 | -0,18 | -0,04 | 0,27 |
| **DP46** | 0,67 | 0,12 | -0,01 | 0,35 | 0,67 | -0,79 | 0,31 | 0,81 | 0,85 | 0,82 | 0,53 | -0,63 | 0,31 | 0,32 | 0,78 | 0,45 | 0,34 | 0,45 | 0,84 | -0,14 | -0,11 | -0,11 | 0,33 |
| **DP47** | 0,65 | 0,00 | -0,13 | 0,24 | 0,59 | -0,71 | 0,19 | 0,87 | 0,91 | 0,89 | 0,44 | -0,69 | 0,19 | 0,22 | 0,85 | 0,37 | 0,26 | 0,34 | 0,85 | -0,25 | 0,01 | -0,17 | 0,44 |
| **DP48** | 0,69 | -0,01 | -0,16 | 0,24 | 0,55 | -0,69 | 0,18 | 0,85 | 0,92 | 0,89 | 0,45 | -0,73 | 0,20 | 0,23 | 0,87 | 0,40 | 0,29 | 0,31 | 0,88 | -0,28 | 0,04 | -0,22 | 0,46 |
| **DP49** | 0,64 | -0,10 | -0,23 | 0,15 | 0,53 | -0,63 | 0,09 | 0,90 | 0,94 | 0,93 | 0,36 | -0,73 | 0,10 | 0,14 | 0,89 | 0,32 | 0,21 | 0,25 | 0,85 | -0,34 | 0,10 | -0,22 | 0,53 |
| **DP50** | 0,65 | -0,14 | -0,29 | 0,12 | 0,47 | -0,59 | 0,05 | 0,90 | 0,96 | 0,95 | 0,34 | -0,77 | 0,07 | 0,11 | 0,92 | 0,31 | 0,20 | 0,19 | 0,86 | -0,40 | 0,16 | -0,27 | 0,58 |
| **DP51** | 0,66 | -0,13 | -0,28 | 0,13 | 0,47 | -0,60 | 0,06 | 0,89 | 0,96 | 0,94 | 0,35 | -0,77 | 0,08 | 0,13 | 0,92 | 0,33 | 0,21 | 0,20 | 0,87 | -0,39 | 0,15 | -0,28 | 0,57 |
| **DP52** | 0,61 | -0,23 | -0,37 | 0,03 | 0,41 | -0,52 | -0,04 | 0,92 | 0,98 | 0,97 | 0,26 | -0,79 | -0,02 | 0,03 | 0,95 | 0,25 | 0,13 | 0,11 | 0,84 | -0,47 | 0,25 | -0,31 | 0,65 |
| **DP53** | 0,69 | -0,15 | -0,32 | 0,13 | 0,42 | -0,57 | 0,04 | 0,87 | 0,97 | 0,95 | 0,35 | -0,82 | 0,07 | 0,14 | 0,94 | 0,35 | 0,24 | 0,15 | 0,89 | -0,44 | 0,20 | -0,34 | 0,60 |
| **DP54** | 0,69 | -0,15 | -0,33 | 0,13 | 0,41 | -0,57 | 0,04 | 0,87 | 0,97 | 0,95 | 0,35 | -0,82 | 0,07 | 0,14 | 0,94 | 0,35 | 0,24 | 0,15 | 0,90 | -0,44 | 0,20 | -0,35 | 0,60 |
| **DP55** | 0,71 | -0,14 | -0,33 | 0,14 | 0,40 | -0,57 | 0,05 | 0,86 | 0,97 | 0,95 | 0,37 | -0,83 | 0,08 | 0,15 | 0,94 | 0,37 | 0,26 | 0,14 | 0,91 | -0,45 | 0,21 | -0,37 | 0,60 |
| **DP56** | 0,73 | -0,15 | -0,36 | 0,13 | 0,36 | -0,55 | 0,03 | 0,85 | 0,97 | 0,95 | 0,37 | -0,85 | 0,07 | 0,15 | 0,95 | 0,38 | 0,27 | 0,11 | 0,92 | -0,47 | 0,24 | -0,41 | 0,62 |
| **DP57** | 0,75 | -0,14 | -0,35 | 0,15 | 0,35 | -0,55 | 0,04 | 0,83 | 0,97 | 0,94 | 0,38 | -0,87 | 0,09 | 0,18 | 0,95 | 0,41 | 0,30 | 0,11 | 0,93 | -0,47 | 0,24 | -0,43 | 0,61 |
| **DP58** | 0,75 | -0,08 | -0,29 | 0,20 | 0,42 | -0,61 | 0,10 | 0,83 | 0,95 | 0,92 | 0,42 | -0,83 | 0,14 | 0,21 | 0,93 | 0,42 | 0,32 | 0,19 | 0,93 | -0,41 | 0,16 | -0,37 | 0,56 |
| **DP59** | 0,77 | -0,09 | -0,32 | 0,20 | 0,37 | -0,58 | 0,09 | 0,81 | 0,96 | 0,92 | 0,43 | -0,86 | 0,14 | 0,22 | 0,94 | 0,44 | 0,34 | 0,15 | 0,94 | -0,44 | 0,20 | -0,42 | 0,57 |
| **DP60** | 0,78 | -0,07 | -0,29 | 0,22 | 0,39 | -0,60 | 0,12 | 0,81 | 0,95 | 0,91 | 0,45 | -0,85 | 0,16 | 0,24 | 0,93 | 0,46 | 0,36 | 0,18 | 0,94 | -0,41 | 0,17 | -0,41 | 0,55 |
| **DP61** | 0,78 | -0,06 | -0,28 | 0,23 | 0,40 | -0,61 | 0,12 | 0,80 | 0,95 | 0,91 | 0,45 | -0,84 | 0,17 | 0,25 | 0,92 | 0,46 | 0,36 | 0,19 | 0,94 | -0,41 | 0,16 | -0,40 | 0,55 |
| **DP62** | 0,78 | 0,00 | -0,22 | 0,28 | 0,46 | -0,66 | 0,19 | 0,80 | 0,93 | 0,89 | 0,50 | -0,81 | 0,22 | 0,29 | 0,89 | 0,49 | 0,39 | 0,25 | 0,94 | -0,34 | 0,09 | -0,35 | 0,49 |
| **DP63** | 0,78 | 0,00 | -0,22 | 0,28 | 0,45 | -0,66 | 0,18 | 0,80 | 0,93 | 0,89 | 0,50 | -0,81 | 0,22 | 0,29 | 0,90 | 0,49 | 0,39 | 0,25 | 0,94 | -0,34 | 0,09 | -0,36 | 0,49 |
| **DP64** | 0,80 | 0,01 | -0,22 | 0,29 | 0,44 | -0,66 | 0,19 | 0,78 | 0,92 | 0,88 | 0,51 | -0,82 | 0,23 | 0,31 | 0,89 | 0,51 | 0,41 | 0,25 | 0,95 | -0,35 | 0,10 | -0,38 | 0,49 |
| **DP65** | 0,78 | 0,05 | -0,16 | 0,32 | 0,50 | -0,71 | 0,24 | 0,78 | 0,91 | 0,87 | 0,53 | -0,78 | 0,27 | 0,33 | 0,87 | 0,51 | 0,41 | 0,31 | 0,93 | -0,29 | 0,04 | -0,31 | 0,44 |
| **DP66** | 0,78 | 0,10 | -0,11 | 0,36 | 0,54 | -0,74 | 0,28 | 0,77 | 0,89 | 0,85 | 0,56 | -0,75 | 0,31 | 0,36 | 0,84 | 0,53 | 0,43 | 0,36 | 0,93 | -0,24 | -0,01 | -0,28 | 0,40 |
| **DP67** | 0,79 | 0,09 | -0,12 | 0,36 | 0,52 | -0,73 | 0,28 | 0,76 | 0,89 | 0,85 | 0,57 | -0,76 | 0,31 | 0,37 | 0,85 | 0,54 | 0,44 | 0,35 | 0,93 | -0,25 | 0,00 | -0,30 | 0,40 |
| **DP68** | 0,79 | 0,02 | -0,20 | 0,30 | 0,46 | -0,68 | 0,21 | 0,78 | 0,92 | 0,88 | 0,52 | -0,81 | 0,25 | 0,31 | 0,89 | 0,51 | 0,41 | 0,27 | 0,94 | -0,33 | 0,08 | -0,36 | 0,47 |
| **DP69** | 0,75 | 0,10 | -0,09 | 0,35 | 0,57 | -0,75 | 0,28 | 0,79 | 0,88 | 0,84 | 0,55 | -0,72 | 0,31 | 0,35 | 0,83 | 0,51 | 0,40 | 0,38 | 0,91 | -0,22 | -0,04 | -0,23 | 0,38 |
| **DP70** | 0,76 | 0,01 | -0,19 | 0,28 | 0,49 | -0,68 | 0,19 | 0,81 | 0,92 | 0,89 | 0,50 | -0,79 | 0,23 | 0,29 | 0,89 | 0,48 | 0,37 | 0,28 | 0,93 | -0,32 | 0,07 | -0,32 | 0,48 |
| **DP71** | 0,75 | 0,08 | -0,11 | 0,33 | 0,56 | -0,74 | 0,26 | 0,80 | 0,89 | 0,86 | 0,54 | -0,73 | 0,29 | 0,33 | 0,84 | 0,49 | 0,38 | 0,36 | 0,91 | -0,23 | -0,02 | -0,24 | 0,40 |
| **DP72** | 0,73 | 0,10 | -0,07 | 0,35 | 0,60 | -0,76 | 0,29 | 0,80 | 0,88 | 0,84 | 0,55 | -0,70 | 0,30 | 0,34 | 0,82 | 0,49 | 0,38 | 0,40 | 0,89 | -0,20 | -0,05 | -0,20 | 0,37 |
| **DP73** | 0,72 | 0,13 | -0,03 | 0,37 | 0,63 | -0,78 | 0,31 | 0,79 | 0,86 | 0,83 | 0,56 | -0,67 | 0,33 | 0,35 | 0,80 | 0,49 | 0,38 | 0,43 | 0,88 | -0,16 | -0,09 | -0,16 | 0,34 |
| **DP74** | 0,65 | 0,23 | 0,11 | 0,43 | 0,75 | -0,86 | 0,41 | 0,76 | 0,78 | 0,75 | 0,59 | -0,54 | 0,40 | 0,39 | 0,70 | 0,48 | 0,37 | 0,56 | 0,80 | -0,01 | -0,23 | -0,01 | 0,21 |
| **DP75** | 0,64 | 0,22 | 0,11 | 0,42 | 0,75 | -0,86 | 0,41 | 0,77 | 0,78 | 0,75 | 0,59 | -0,54 | 0,39 | 0,38 | 0,70 | 0,47 | 0,36 | 0,56 | 0,80 | -0,01 | -0,24 | 0,00 | 0,21 |
| **DP76** | 0,64 | 0,21 | 0,10 | 0,41 | 0,74 | -0,85 | 0,39 | 0,78 | 0,79 | 0,76 | 0,58 | -0,54 | 0,38 | 0,37 | 0,71 | 0,46 | 0,36 | 0,55 | 0,80 | -0,02 | -0,22 | -0,01 | 0,23 |
| **DP77** | 0,64 | 0,22 | 0,11 | 0,42 | 0,75 | -0,85 | 0,40 | 0,77 | 0,78 | 0,75 | 0,58 | -0,54 | 0,39 | 0,38 | 0,70 | 0,47 | 0,36 | 0,56 | 0,80 | -0,01 | -0,23 | 0,00 | 0,22 |
| **DP78** | 0,63 | 0,19 | 0,08 | 0,39 | 0,74 | -0,84 | 0,38 | 0,79 | 0,80 | 0,77 | 0,56 | -0,55 | 0,36 | 0,35 | 0,72 | 0,44 | 0,34 | 0,54 | 0,80 | -0,03 | -0,21 | -0,01 | 0,24 |
| **DP79** | 0,61 | 0,16 | 0,07 | 0,36 | 0,74 | -0,82 | 0,35 | 0,81 | 0,81 | 0,78 | 0,53 | -0,55 | 0,33 | 0,32 | 0,72 | 0,41 | 0,30 | 0,52 | 0,79 | -0,05 | -0,19 | 0,00 | 0,26 |
| **DP80** | 0,63 | 0,19 | 0,09 | 0,39 | 0,75 | -0,84 | 0,37 | 0,79 | 0,80 | 0,77 | 0,55 | -0,54 | 0,36 | 0,34 | 0,71 | 0,44 | 0,33 | 0,54 | 0,79 | -0,03 | -0,21 | 0,00 | 0,24 |
| **DP81** | 0,62 | 0,16 | 0,06 | 0,36 | 0,73 | -0,82 | 0,35 | 0,81 | 0,81 | 0,79 | 0,53 | -0,56 | 0,33 | 0,32 | 0,73 | 0,42 | 0,31 | 0,51 | 0,80 | -0,06 | -0,18 | -0,01 | 0,27 |
| **DP82** | 0,63 | 0,17 | 0,07 | 0,37 | 0,73 | -0,83 | 0,36 | 0,80 | 0,81 | 0,78 | 0,54 | -0,56 | 0,34 | 0,33 | 0,73 | 0,43 | 0,32 | 0,52 | 0,80 | -0,05 | -0,19 | -0,01 | 0,26 |
| **DP83** | 0,62 | 0,16 | 0,06 | 0,37 | 0,73 | -0,82 | 0,35 | 0,81 | 0,81 | 0,78 | 0,54 | -0,55 | 0,34 | 0,32 | 0,73 | 0,42 | 0,31 | 0,52 | 0,79 | -0,05 | -0,19 | -0,01 | 0,27 |
| **DP84** | 0,64 | 0,16 | 0,04 | 0,37 | 0,72 | -0,82 | 0,34 | 0,81 | 0,82 | 0,79 | 0,54 | -0,58 | 0,33 | 0,33 | 0,74 | 0,44 | 0,33 | 0,50 | 0,81 | -0,07 | -0,17 | -0,04 | 0,28 |
| **DP85** | 0,60 | 0,15 | 0,06 | 0,35 | 0,74 | -0,82 | 0,34 | 0,82 | 0,81 | 0,78 | 0,52 | -0,54 | 0,32 | 0,31 | 0,73 | 0,40 | 0,29 | 0,52 | 0,78 | -0,05 | -0,19 | 0,01 | 0,27 |
| **DP86** | 0,61 | 0,14 | 0,05 | 0,35 | 0,73 | -0,81 | 0,33 | 0,82 | 0,82 | 0,79 | 0,52 | -0,56 | 0,32 | 0,30 | 0,74 | 0,40 | 0,29 | 0,51 | 0,79 | -0,07 | -0,18 | -0,01 | 0,28 |
| **DP87** | 0,63 | 0,16 | 0,05 | 0,36 | 0,73 | -0,82 | 0,35 | 0,81 | 0,81 | 0,79 | 0,54 | -0,56 | 0,33 | 0,32 | 0,74 | 0,43 | 0,32 | 0,51 | 0,80 | -0,06 | -0,18 | -0,02 | 0,27 |
| **DP88** | 0,63 | 0,14 | 0,03 | 0,35 | 0,71 | -0,81 | 0,33 | 0,82 | 0,83 | 0,80 | 0,52 | -0,58 | 0,31 | 0,31 | 0,75 | 0,42 | 0,31 | 0,49 | 0,80 | -0,08 | -0,16 | -0,03 | 0,29 |
| **DP89** | 0,65 | 0,13 | 0,02 | 0,35 | 0,70 | -0,80 | 0,32 | 0,82 | 0,84 | 0,81 | 0,53 | -0,60 | 0,32 | 0,31 | 0,76 | 0,43 | 0,32 | 0,48 | 0,82 | -0,10 | -0,14 | -0,06 | 0,31 |
| **DP90** | 0,65 | 0,15 | 0,03 | 0,37 | 0,71 | -0,81 | 0,34 | 0,81 | 0,83 | 0,80 | 0,54 | -0,59 | 0,33 | 0,33 | 0,75 | 0,44 | 0,33 | 0,49 | 0,82 | -0,09 | -0,16 | -0,05 | 0,29 |
| **DP91** | 0,62 | 0,15 | 0,06 | 0,36 | 0,73 | -0,82 | 0,34 | 0,81 | 0,81 | 0,79 | 0,53 | -0,55 | 0,33 | 0,31 | 0,73 | 0,41 | 0,30 | 0,51 | 0,79 | -0,06 | -0,18 | -0,01 | 0,27 |
| **DP92** | 0,62 | 0,19 | 0,10 | 0,39 | 0,75 | -0,84 | 0,38 | 0,79 | 0,79 | 0,76 | 0,55 | -0,53 | 0,36 | 0,34 | 0,71 | 0,43 | 0,32 | 0,55 | 0,78 | -0,02 | -0,22 | 0,01 | 0,23 |
| **DP93** | 0,63 | 0,17 | 0,06 | 0,37 | 0,73 | -0,83 | 0,35 | 0,80 | 0,81 | 0,78 | 0,54 | -0,56 | 0,34 | 0,33 | 0,73 | 0,43 | 0,32 | 0,52 | 0,80 | -0,06 | -0,19 | -0,02 | 0,27 |
| **DP94** | 0,65 | 0,16 | 0,04 | 0,37 | 0,71 | -0,82 | 0,35 | 0,80 | 0,82 | 0,79 | 0,55 | -0,58 | 0,34 | 0,33 | 0,74 | 0,44 | 0,33 | 0,50 | 0,82 | -0,07 | -0,17 | -0,04 | 0,28 |
| **DP95** | 0,63 | 0,18 | 0,08 | 0,39 | 0,74 | -0,84 | 0,37 | 0,79 | 0,80 | 0,77 | 0,56 | -0,55 | 0,36 | 0,34 | 0,72 | 0,44 | 0,33 | 0,53 | 0,80 | -0,04 | -0,20 | -0,01 | 0,25 |
| **DP96** | 0,63 | 0,15 | 0,04 | 0,36 | 0,72 | -0,81 | 0,34 | 0,81 | 0,82 | 0,80 | 0,53 | -0,58 | 0,32 | 0,32 | 0,75 | 0,43 | 0,32 | 0,50 | 0,81 | -0,08 | -0,17 | -0,03 | 0,29 |
| **DP97** | 0,61 | 0,18 | 0,09 | 0,38 | 0,75 | -0,84 | 0,37 | 0,80 | 0,79 | 0,77 | 0,54 | -0,53 | 0,35 | 0,33 | 0,71 | 0,42 | 0,31 | 0,54 | 0,78 | -0,03 | -0,21 | 0,02 | 0,24 |
| **DP98** | 0,62 | 0,18 | 0,08 | 0,38 | 0,75 | -0,83 | 0,37 | 0,80 | 0,80 | 0,77 | 0,54 | -0,54 | 0,35 | 0,33 | 0,71 | 0,43 | 0,32 | 0,54 | 0,79 | -0,03 | -0,21 | 0,01 | 0,25 |
| **DP99** | 0,63 | 0,11 | 0,00 | 0,33 | 0,69 | -0,79 | 0,30 | 0,83 | 0,84 | 0,82 | 0,51 | -0,60 | 0,29 | 0,29 | 0,77 | 0,41 | 0,30 | 0,46 | 0,81 | -0,12 | -0,13 | -0,06 | 0,32 |
| **DP100** | 0,69 | 0,12 | -0,02 | 0,35 | 0,66 | -0,79 | 0,31 | 0,81 | 0,85 | 0,82 | 0,54 | -0,64 | 0,31 | 0,33 | 0,79 | 0,4 | 0,35 | 0,45 | 0,85 | -0,14 | -0,11 | -0,12 | 0,34 |
