# Supplementary material for: Intra-Sample Heterogeneity of Potato Starch Reveals Fluctuation of Starch-Binding Proteins According to Granule Morphology
Source: Plants (Basel). 2019 Sep 4;8(9):324. doi: 10.3390/plants8090324 (PMC6784226; doi:10.3390/plants8090324)
Supplement: Supplementary file 1 [file plants-08-00324-s001.zip › Sup/Table S3.docx]

**Table S3.** Pearson paired-samples correlation analysis between starch-bound protein concentrations and phosphate contents or granule morphology. The average protein concentrations in fmol.mg^-1^. Positive correlations with ρ > 0.90 and *p* < 0.1, ρ > 0.98 and *p* < 0.02, or ρ > 0.99 and *p* < 0.01 are highlighted in red. Significant negative correlation are highlighted in yellow with the use of the same thresholds.

|  | **GBSS** | **SS2** | **LESV** | **LSF2** | **THRx** | **GWD** | **SS3** | **CYP20.2** | **BE1.1** | **BE1.2** | **BE2** | **ESV1** | **PTST1** | **SS1** | **GPx** | **SEX4** | **SEX4.like** | **PHS1a** | **ISA3** | **PWD** | **SS4** | **PHS1b** | **SS6** |
| --- | --- | --- | --- | --- | --- | --- | --- | --- | --- | --- | --- | --- | --- | --- | --- | --- | --- | --- | --- | --- | --- | --- | --- |
| **Total-P (‰)** | -0,61 | -0,56 | -0,46 | -0,69 | -0,87 | 0,99 | -0,71 | -0,50 | -0,50 | -0,45 | -0,78 | 0,28 | -0,68 | -0,63 | -0,40 | -0,62 | -0,53 | -0,82 | -0,66 | -0,35 | 0,57 | -0,19 | 0,16 |
| **C3-P (‰)** | -0,61 | -0,35 | -0,26 | -0,52 | -0,83 | 0,92 | -0,53 | -0,69 | -0,68 | -0,64 | -0,65 | 0,42 | -0,50 | -0,46 | -0,58 | -0,51 | -0,41 | -0,68 | -0,74 | -0,14 | 0,38 | -0,11 | -0,07 |
| **C6-P (‰)** | -0,61 | -0,59 | -0,49 | -0,71 | -0,87 | 0,99 | -0,73 | -0,47 | -0,47 | -0,42 | -0,80 | 0,26 | -0,70 | -0,65 | -0,37 | -0,63 | -0,55 | -0,83 | -0,65 | -0,37 | 0,59 | -0,20 | 0,19 |
| **Diameter (µm)** | 0,69 | 0,42 | 0,28 | 0,60 | 0,79 | -0,94 | 0,58 | 0,62 | 0,65 | 0,61 | 0,73 | -0,45 | 0,57 | 0,55 | 0,57 | 0,60 | 0,51 | 0,69 | 0,78 | 0,16 | -0,40 | 0,04 | 0,03 |
| **Ellipsoid roundness** | -0,75 | -0,68 | -0,48 | -0,83 | -0,74 | 0,98 | -0,80 | -0,33 | -0,42 | -0,35 | -0,91 | 0,33 | -0,81 | -0,80 | -0,35 | -0,80 | -0,74 | -0,80 | -0,72 | -0,36 | 0,58 | -0,03 | 0,23 |
